# Investigation of the Systemic Immune Inflammation (SII) Index as an Indicator of Morbidity and Mortality in Type 2 Diabetic Retinopathy Patients in a 4-Year Follow-Up Period

**DOI:** 10.3390/medicina60060855

**Published:** 2024-05-24

**Authors:** Nilgun Tan Tabakoglu, Mehmet Celik

**Affiliations:** 1 Health Research and Development Center, Faculty of Medicine Hospital, Trakya University, Edirne 22100, Turkey; 2 Department of Endocrine and Metabolic Diseases, Faculty of Medicine, Trakya University, Edirne 22100, Turkey; mehmetcelik@trakya.edu.tr

**Keywords:** systemic immune inflammation, diabetic retinopathy, type 2 diabetes mellitus, microvascular complications, macrovascular complications

## Abstract

*Background and Objectives*: This study aimed to investigate the relationship between the systemic immune inflammation (SII) index and the development of micro and macro complications and mortality within the first year and the following three years in type 2 diabetic retinopathy patients. *Materials and Methods*: The retrospective study included 523 type 2 diabetic retinopathy patients seen in the endocrinology outpatient clinic of our hospital between January and December 2019. Their demographic and clinical characteristics were analyzed using descriptive statistics. The normal distribution of quantitative data was assessed by the Shapiro–Wilk test. Mann–Whitney U, McNemar–Chi-square, and Cochran’s Q tests were used to analyze the SII values and complication rates over time. An ROC analysis determined the sensitivity and specificity of SII. A multiple linear regression analysis examined the relationship between variables and SII, while Spearman’s test assessed the correlation between CRP and SII. *p* < 0.05 was accepted as significant. *Results*: The mean age of patients was 63.5 ± 9.3 years, with mean SII values of 821.4 ± 1010.8. Higher SII values were significantly associated with acute–chronic renal failure, peripheral arterial disease, and hospitalization rates in both the first year and the following three years (*p* < 0.05 for all). Significant cut-off values for SII were found for micro- and macrovascular complications and death within the first year (*p* < 0.05 for all). The ROC curve analysis identified an optimal SII cut-off value of >594.0 for predicting near-term (1-year) complications and mortality, with a sensitivity of 73.8% and specificity of 49.4% (area under the ROC curve: 0.629, *p* = 0.001). Multiple linear regression indicated that smoking of at least 20 pack-years had a significant positive effect on SII. The Spearman test showed a weak positive correlation between SII and CRP. *Conclusions*: High SII values predict both early and late acute–chronic renal failure, peripheral arterial disease, and hospitalizations in patients with type 2 diabetic retinopathy. The study also shows that high SII values may predict microvascular and macrovascular complications of type 2 DM and mortality risk in the early period in patients with type 2 diabetic retinopathy. In addition, comorbidities and inflammatory habits, such as long-term smoking, should be considered in the clinical use of SII.

## 1. Introduction

Diabetes mellitus (DM) is a chronic metabolic disease characterized by hyperglycemia and complications caused by impairments in any or all of the production, release, and effects of insulin, a peptide hormone secreted by the β cells of the islets of Langerhans of the pancreas [1]. It has been reported that 90% of diabetes cases in the world are type 2 DM cases [2]. According to the *Diabetes Atlas* published periodically by the International Diabetes Federation, the number of people diagnosed with diabetes globally by the end of 2021 was recorded as 537 million. Even more remarkably, this number is estimated to reach 783 million by 2045 [3]. Chronic complications of type 2 DM are categorized under two main headings as micro- and macrovascular complications: nephropathy, retinopathy, and neuropathy are defined as microvascular complications, while coronary artery disease (CAD), peripheral arterial disease (PAD), and cerebrovascular disease (CVD) are defined as macrovascular complications [4,5]. Chronic hyperglycemia is a critical factor in the formation of these complications. The duration of diabetes and comorbidities are other factors affecting the development of complications. Diabetic retinopathy (DR) is the most common microvascular complication and is a precursor of other microvascular complications. One out of every three patients with type 2 DM develops DR. It is also the most common cause of preventable new cases of blindness in people aged 20–74 years [6].

Fundus examination is critical to detect the first signs of DR. Initial signs include damage to the endothelium of the retinal vasculature, microaneurysms, hemorrhage, and exudate development. With DR progression, capillary occlusion, neovascularization, and subsequent retinal detachment may develop. Retinal lesions are detected with an ophthalmoscope. Retinal lesions are divided into two types according to their intensity: non-proliferative diabetic retinopathy (NPDR) and proliferative diabetic retinopathy (PDR). Fundus fluorescein angiography (FFA) and optical coherence tomography (OCT) are used for diagnosis and follow-up. These are costly, invasive, and specialized methods. At the same time, each of the methods used to monitor the development of other micro and macro complications in patients with type 2 DM increases the financial burden. Therefore, it is important to have additional biomarkers for the diagnosis and follow-up of both DR and other complications in type 2 DM patients with DR in many peripheral regions.

Hyperglycemia-induced glycosylated proteins damage cells, causing dysfunction and initiating the inflammatory process by inducing the release of tumor necrosis factor-alpha (TNF-α), free radicals, interleukin-6 (IL-6), and C-reactive protein (CRP). This is recognized as one of the main mechanisms of vascular damage, which is the cause of the chronic complications of diabetes [2].

During the inflammatory response, platelets mediate the activation of circulating leukocytes and their adhesion to the endothelial surface, leading to both changes in the number of circulating leukocytes and endothelial damage [7].

The determination of systemic immune inflammation (SII) cut-off values in patients with DR may be a warning for the prevention of other complications and mortality.

SII derived from laboratory parameters has recently come to the forefront in determining the prognosis of many diseases. SII, which can be calculated simply by using platelet, lymphocyte, and neutrophil values from complete blood count (CBC) values, was first developed in 2014 to predict survival rates of individuals with hepatocellular carcinoma [8,9].

There is increasing evidence that there may be an association between SII and metabolic derangements and their components [10]. Moreover, considering that macro- and microvascular complications of type 2 DM have chronic inflammation and metabolic derangements as common risk factors, it is a valid assumption that people with high SII levels have a higher risk of these complications.

Numerous studies have examined the relationship between type 2 DM, its complications, and SII. However, no study has addressed the association of SII with duration in predicting macrovascular and other microvascular complications and mortality rates in type 2 diabetic retinopathy patients.

This study was conducted to fill the existing literature gap and examine the relationship between SII levels, complications, and mortality rates in type 2 diabetic retinopathy patients during the first year of follow-up and the subsequent three years after the index was calculated.

## 2. Materials and Methods

This study was designed as a single-center and retrospective study. Between January and December 2019, 523 patients over 18 years old who were admitted to the endocrinology outpatient clinic of our hospital with a DR diagnosis of type 2 DM were included in the study. Patients diagnosed with type 1 DM, patients with acute infectious disease, sepsis, chronic inflammatory and rheumatologic diseases, active malignancies, other endocrinologic disorders, patients under 18 years of age, pregnant women, and breastfeeding women were excluded. For this study, TÜTF-GOBAEK 2023/341 approval was received from the ethics committee of our Faculty of Medicine, dated 29 September 2023. DR progression was defined as increased macular edema, decreased visual acuity, and the conversion of NPDR to PDR.

Demographic information, laboratory data at the time of admission, medical history, and examination findings of the patients included in the study were obtained from the patient file records of the hospital. The demographic information of the patients was determined as age, gender, alcohol, and smoking. The diagnosis of DR was confirmed according to the EURETINA guidelines, and type 2 DM was confirmed according to the American Diabetes Association 2019 criteria [11,12]. The medical history of the patients included the duration of DM, a diagnosis and duration of hypertension, a diagnosis of heart failure, a diagnosis of cerebrovascular disease, insulin use, and antitriglyceridemic and antihyperlipidemic drug use. Laboratory parameters in the study were measured in the Biochemistry Department of our hospital using an automated analyzer. The SII value was obtained using the following: platelet count at admission x neutrophil count/lymphocyte count ratio [13,14]. Macro and micro complications and clinical findings of the patients during follow-up were obtained through outpatient clinic records, phone calls, and an e-pulse system [15].

Descriptive statistics regarding the demographic and clinical characteristics of patients were shown as mean, standard deviation, number, and %. The suitability of quantitative data for normal distribution was examined with the Shapiro–Wilk test. The Mann–Whitney U test was used to compare SII values between groups. The McNemar–Chi-square test was used in the comparison of complication rates between the first year and the following three years. The Related-Samples Cochran’s Q test was used in the comparison of complication rates between the baseline, first year, and the following three years. Using the Receiver Operating Characteristic (ROC) analysis method and the area under the curve (AUC), the cut-off points and the sensitivity and specificity values were calculated for SII. The relationship between SII and the variables hypertension, at least 20 pack-years of smoking, antihyperlipidemic/antitriglyceridemic drug use, DM insulin usage, and gender was examined using multiple linear regression analysis. The relationship between CRP and SII was examined using the Spearman test. *p* < 0.05 value was accepted as statistical significance. The SPSS 20.0 package program (IBM SPSS Statistics for Windows, Version 20.0. Armonk, NY, USA: IBM Corp.) was used to analyze the data.

## 3. Results

The demographic and clinical characteristics of the patients are shown in Table 1. The average age of the patients was 63.5 ± 9.3 years, and 40.3% were women. The average SII values were found to be 821.4 ± 1010.8.

The rate of polyneuropathy was 39.4% in the first year and 43.3% in the following three years. Retinopathy progression was detected as 20.3% in the first year and 28.8% in the following three years. While the rate of microvascular complications was 74.6% in the first year, this rate decreased to 61.8% in following three years (*p* < 0.001). While the rate of macrovascular complications was 45.9% in the first year, this rate decreased to 30.4% in following three years (*p* < 0.001). Similarly, a significant decrease was observed in the rates of micro- and macrovascular complications, microvascular complications and death, macrovascular complications and death, and micro- and macrovascular complications and death combinations in following three years (*p* < 0.001; Table 2).

The comparison of patients’ SII values in the first year and the following three years according to the presence of micro and macro complications, hospitalization for any reason, and hospitalization for DM parameters is shown in Table 3. Accordingly, the SII values of those with acute–chronic renal failure, peripheral artery disease, and hospitalization were found to be significantly higher both in the first year and following three years (*p* < 0.05 for all). No significant difference was found in the SII values in terms of Polyneuropathy, CAD, Retinopathy Progression, and hospitalization due to DM (*p* > 0.05 for all).

The comparison of SII values in the first year and the following three years according to micro- and macrovascular complications and death is shown in Table 4. Accordingly, in the first year, SII values were found to be significantly higher in those with microvascular and macrovascular complications, in those who died, and in combinations of these parameters (*p* < 0.05 for all). In following three years, no significant difference was found in the SII values in terms of micro- and macrovascular complications and death (*p* > 0.05 for all).

The results of the ROC analysis of SII values regarding micro- and macrovascular complications and death are shown in Table 5. Accordingly, the area under the curve (AUC) values in the first year were 0.608 (*p* < 0.001) for microvascular complications, 0.570 (*p* = 0.005) for macrovascular complications, 0.618 (*p* < 0.001) for death, 0.589 (*p* < 0.001) for micro- and macrovascular complications, and 0.629 (*p* = 0.001) for micro- and macrovascular complications and death. The area under the curve values in the following three years were not significant (*p* > 0.05 for all).

**Figure 1:**
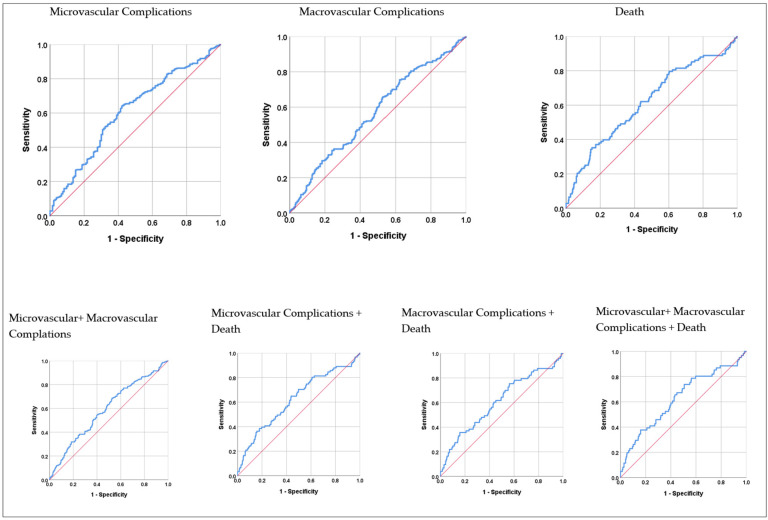
ROC analysis results of SII values for microvascular and macrovascular complications and death in the first year. Blue line: Represents the performance of the SII, showing the balance between sensitivity and specificity; Red line: Represents a random classifier with no discrimination ability and an AUC of 0.5.

The results of the ROC analysis of the SII values regarding micro- and macrovascular complications and death in the first year are shown graphically in Figure 1. It was found that the SII values reached the highest diagnostic accuracy rate in the first year in the micro- and macrovascular complications and death combination, and at the cut-off point calculated as >594.0, the sensitivity value was 73.8%, and the specificity value was 49.4%.

A multiple linear regression analysis was conducted to investigate the effects of various factors on SII. The model included hypertension, smoking (at least 20 pack-years), antihyperlipidemic and antitriglyceridemic drug use, DM insulin usage, and gender as predictors. The overall model was not statistically significant (F(7, 510) = 1.065, *p* = 0.385), indicating that these factors collectively did not explain a significant portion of the variance in SII (R^2^ = 0.014, Adjusted R^2^ = 0.001).

Among the predictors, only at least 20 pack-years of smoking showed a statistically significant positive effect on SII (B = 180.053, *p* = 0.034, Table 6). The other factors, including hypertension, antihyperlipidemic and antitriglyceridemic medication use, DM insulin usage, and gender, did not show a significant relationship with SII (*p* > 0.05 for all, Table 6).

The Spearman correlation analysis showed a weak positive correlation between SII and CRP (r = 0.266; *p* < 0.001; Table 7).

## 4. Discussion

This study investigated the relationship between the predictive value of SII on other microvascular complications of type 2 DM (nephropathy and neuropathy), macrovascular complications (coronary artery disease, peripheral artery disease, and cerebrovascular disease), and mortality rates in patients diagnosed with DR, which is one of the most common microvascular complications of type 2 DM, in early and late periods (the first year and the following three years). The findings of our study show that high SII values were associated with increased mortality and the development of other microvascular and macrovascular complications and were statistically significant in predicting the likelihood of mortality and the development of complications, especially in the first year following the calculation of the SII value.

Type 2 DM is a chronic disease characterized by persistent hyperglycemia. Due to hyperglycemia, glucose reacts non-enzymatically with lipids and proteins, releasing advanced glycation end products (AGEs) and causing oxidative stress. The resulting oxidative stress increases the levels of reactive oxygen species (ROS), leading to impaired blood flow in small blood vessels and impaired tissue nutrition, leading to microvascular complications. In large blood vessels, it facilitates the development of atherosclerotic plaques, which narrow the arteries and restrict blood flow, leading to macrovascular complications [16,17]. Sustained hyperglycemia triggers the immune response, leading to the synthesis of pro-inflammatory cytokines, C-reactive protein, tumor necrosis factor (TNF)-α, interleukin-6 (IL-6), and chemokines [18]. Chronic inflammation disrupts the structure of small vessels and contributes to the development of microvascular complications. In large vessels, they enter atherosclerotic plaques and destabilize them. They cause plaque ruptures [19]. Endothelial dysfunction develops. Another condition that contributes to endothelial dysfunction is adipokines (adiponectin, leptin, and resistin). Adiponectin is anti-inflammatory, whereas leptin and resistin are inflammatory. Adiponectin increases nitric oxide (NO) levels. However, in type 2 DM, adiponectin is decreased, which leads to a decrease in the NO level and an increase in the endothelin-1 level, which disrupts the balance in small vessels in favor of vasoconstriction and leads to the development of microvascular complications. In large vessels, endothelial dysfunction plays an important role in the emergence of macrovascular complications by facilitating vasoconstriction, inflammation, and oxidative stress [20].

DR is a microvascular complication of diabetes in the eye with an increasing prevalence. Once it occurs, it is irreversible. It can progress to vision loss. Current treatments aim to halt its progression. Chronic minimal inflammation plays a role in its pathogenesis. An animal model study in diabetic mice showed that the progression of DR was slowed when inflammatory pathways were blocked [21]. Another study examined the levels of inflammatory proteins in patients with DR using proteomic techniques and found an increase in these levels [22]. These studies demonstrate the role of chronic minimal inflammation in the development and progression of DR and suggest that the inhibition of inflammation may prevent the progression of the disease.

Many inflammatory cells are involved in developing and maintaining chronic minimal inflammation. The most prominent feature of this inflammatory process is the increased permeability of the vascular wall secondary to inflammation, allowing inflammatory cells to pass from the vessels to the tissues. Neutrophils are the first cells to arrive when inflammation occurs. Under chronic inflammation, neutrophils are constantly activated, leading to the formation and accumulation of neutrophil extracellular traps (NETs). This leads to vascular occlusion, tissue damage, and the exacerbation of inflammation, increasing damage at the site of inflammation. In a study comparing the neutrophil levels of patients with type 2 DM and healthy individuals, it was found that the neutrophil levels of the patient group were significantly higher [22]. Significant increases in the number of neutrophil, monocyte–macrophage, and platelet cells have been recorded in type 2 DM patients during the inflammation process. In a systematic meta-analysis study examining the changes in hematologic parameters of patients with type 2 DM and type 1 DM and a control group of healthy individuals, it was shown that patients with type 2 DM had significantly higher absolute neutrophil, absolute monocyte, absolute lymphocyte, and absolute basophil counts and relative neutrophil and basophil counts [23]. In addition, another study reported that hematologic parameters, such as the neutrophil-to-lymphocyte ratio (NLR) and monocyte-to-platelet ratio, were higher in patients with diabetes and that these increases could be considered a sign of inflammatory response [24]. In another study, the neutrophil levels of patients with type 2 DM and healthy individuals were compared, and it was found that the neutrophil levels of the patient group were significantly higher [25]. In another study, it was concluded that NET levels were elevated in type 2 DM patients and that NETs play a role in the pathogenesis of both DM and microvascular complications, especially diabetic nephropathy and cardiac disease associated with DM [26]. One of the other cells involved in chronic minimal inflammation is monocyte–macrophages, which maintain and exacerbate the inflammatory process by secreting pro-inflammatory cytokines (IL-1β, TNF-α, IL-6, IL-8, MCP-1, and IL-1) [27]. Another cell is platelets, which both form thrombosis with their own activation and increase their activation by binding to leukocyte subcellular groups [28]. An increase in neutrophil, monocyte–macrophage, and platelet cell numbers indicates inflammation. These results are compatible with our study because the neutrophil, monocyte, and platelet values were found to be high in patients with DR in our study (SII: 821.4 ± 1010.8; Table 1).

SII, one of the most frequently used inflammatory markers in recent years, is less affected by physiopathologic changes. This makes SII more reliable and more sensitive in showing existing inflammation [29]. Other advantages are that it is cost-effective and can be calculated with hemogram results that can be performed even in peripheral areas. In a study of 500 type 2 DM patients with and without DR, SII was found to be an inflammatory marker that can be used for early diagnosis of DR [30]. In our study, our patients had a diagnosis of DR, but we found that SII had no predictive value in DR progression in the first year and the following three years of follow-up (retinopathy progression *p* > 0.05; Table 3). This may be because the SII values of all patients already diagnosed with DR were higher than the SII of only patients with type 2 DM due to inflammation. In a study in which 100 uncomplicated type 2 DM patients were included, and many biomarkers were examined, the mean SII value was found to be 470.91 [31]. In our study, all of our patients were type 2 DM patients with DR diagnosis, and the mean value for SII was found to be 821.4. Due to the existing microvascular complications, the mean SII value in our study was almost twice as high compared to uncomplicated type 2 DM in the other study. For this reason, chronic minimal inflammation may have continued in the following years but may not have reached very high levels with its progression. Another reason may be that 108 patients died within one year after the calculated SII values, and 29 patients died within the following three years (Table 2). As a result, we do not know how many of these deceased patients had DR progression. In a study of 300 type 2 DM patients on whether SII has a predictive value for diabetic nephropathy and cardiovascular diseases in patients with type 2 DM, predictive SII values were found for cardiovascular diseases and diabetic nephropathy [31]. In a study investigating the relationship between SII and diabetic neuropathy in 1460 hospitalized type 2 DM patients in China, it was found that SII levels were significantly higher in patients with diabetic neuropathy [32]. In another study in which 584 patients with diabetic nephropathy (DN) due to type 2 DM, type 2 DM patients without DN, and a control group were investigated for the relationship between SII and DN, it was found that high SII levels were associated with DN [33]. The results of our study are consistent with the literature. SII was found to be statistically significant in predicting type 2 diabetic retinopathy patients to be diagnosed with acute or chronic renal failure due to diabetic nephropathy in the first year and the following three years, the development of peripheral arterial disease, and the hospitalization of patients for any reason (Table 3). It was not statistically significant in predicting coronary artery disease, hospitalization due to diabetes, and the development of polyneuropathy both in the first year and the following three years (Table 3). This may be due to the fact that the basal inflammation was high in those who died, and chronic minimal inflammation was ongoing due to DR, and therefore, the patient’s immune systems were active, or other microvascular complications may have started due to the development of DR but were not detected because they were not yet diagnosed. In addition, it has been observed that type 2 diabetic retinopathy patients are more likely to be hospitalized due to complications. As patients who develop micro- and macrovascular complications are mostly patients with a poor glycemic index, who do not pay attention to their diet and do not follow the controls, it is natural that they are frequently hospitalized due to complications that develop as a result. Another reason may be the need for a larger sample group, which is also one of the limitations of our study.

One of the most important results of this study is that SII values have significant predictive power for parameters, such as micro- and macrovascular complications and death and combinations of these parametrics in the early period, but this predictive power decreases with increasing duration. In the first year, the significant association between high SII values and these clinical outcomes (*p* < 0.05; Table 4) suggests that SII may be a valuable biomarker for short-term risk assessment. However, the lack of a significant difference supporting this association over the following three years (*p* > 0.05; Table 4) suggests that the long-term clinical predictive value of SII is limited. This finding emphasizes the need for further investigation of the dynamic changes in inflammatory markers over time and their impact on long-term clinical outcomes.

The focus of our study was to examine the relationship between the SII values of patients with type 2 diabetic retinopathy and the development of micro- and macrovascular complications and death in the first year and the following three years. When the SII values of the patients were analyzed by an ROC analysis for microvascular complications, macrovascular complications, death, micro- and macrovascular complications, microvascular complications and death, macrovascular complications and death, micro- and macrovascular complications and death, and micro- and macrovascular complications and death (composite endpoint) for the first year and following three years, a statistically significant optimal SII value was confirmed for all parameters in the first year (*p* < 0.05; Table 5). For following three years, the SII values determined were not statistically significant (*p* > 0.05; Table 5). This may be due to the relationship between the existing inflammation and duration. In general, our SII values were found to be high because type 2 DM, DR, and its complications are based on chronic minimal inflammation, and SII is an inflammatory marker. This is consistent with the literature. The ROC curve analysis confirmed that the optimal SII value for the composite endpoint, >594.0, predicted the composite endpoint with a higher sensitivity of 73.8%, specificity of 49.4%, and AUC= 0.629 compared to other parameters (*p* = 0.001; Table 5; Figure 1).

We applied multiple linear regression analysis to determine whether the variables in our study affected the SII values. We found that only “at least 20 pack-years of smoking” had a statistically significant positive effect on SII (B = 180.053, *p* = 0.034). This indicates that individuals who have smoked for at least 20 pack-years have higher levels of systemic immune inflammation. This finding is consistent with previous studies linking heavy smoking to increased inflammatory responses [34]. In contrast, (B = −132.996, *p* = 0.292), the use of antihyperlipidemic and antitriglyceridemic drugs (B = 2.806, *p* = 0.967), use of DM insulin (B = 11.876, *p* = 0.861), and gender (B = −22.822, *p* = 0.758) did not show statistically significant relationships with SII. These results suggest that these factors do not have a significant impact on systemic immune inflammation in this study population. This may be due to various reasons, such as the sample size not being large enough to detect subtle effects, or these variables affect SII through mechanisms not captured in this analysis. Further research is needed to better understand and manage conditions associated with inflammation by exploring other potential determinants and mechanisms affecting SII.

Another inflammation biomarker, CRP, is known to rise in response to inflammation in the body and is widely used. In a 2015 meta-analysis study examining the relationship between CRP and diabetic retinopathy, it was concluded that CRP could be used as a biomarker to determine the severity of diabetic retinopathy [35]. Given that SII is an inflammatory index, we examined the relationship between CRP values and SII values in our patients using the Spearman test. Consistent with the above literature, we found a weak positive relationship between the two inflammatory markers (r = 0.266; *p* < 0.001; Table 7). However, we did not find any studies in the literature regarding CRP’s ability to predict early and late-term mortality and morbidity in patients with type 2 diabetic retinopathy.

This study found that the SII index is an effective predictor of morbidity and mortality in patients with type 2 diabetic retinopathy. High SII values were significantly associated with acute and chronic renal failure, peripheral arterial disease and hospitalization rates. These findings suggest that the SII index can be used to predict microvascular and macrovascular complications and mortality risk in patients with type 2 diabetic retinopathy, especially within 1 year of calculating SII values. However, combining SII with other biomarkers and artificial intelligence methods may improve diagnostic accuracy. Integrating various biomarkers and AI methods has shown potential to improve diagnostic processes in clinical applications, as demonstrated in studies involving hemogram-based decision tree models to differentiate COVID-19 patients [36]. Similarly, the use of additional biomarkers and AI in combination with SII may improve the long-term prediction of complications and mortality risk in type 2 diabetic retinopathy patients.

Our study had several limitations, the most important of which was that it was a retrospective and single-center study. Another one is the small number of participants. Moreover, the specificity values of the SII are low; therefore, they should be carefully interpreted in their clinical usage.

## 5. Conclusions

Our research is significant in determining the predictive cut-off value of the SII for microvascular and macrovascular complications, mortality, and composite endpoints in the first year among patients with type 2 diabetic retinopathy. Additionally, both in the first year and the following three years, it is valuable for identifying SII values that can predict the development of acute or chronic renal failure due to diabetic nephropathy, peripheral arterial disease, and hospital admissions for any reason among these patients. This capability enables the early diagnosis of potential complications in patients with type 2 diabetic retinopathy, leading to timely treatment. Thus, patients at risk can be identified through simple hemogram parameters used to calculate the SII, potentially preventing or halting the progression of diabetic complications. This not only helps prevent a decline in patients’ quality of life but also results in significant healthcare cost savings. Moreover, predicting the risk of death within the first year could also enhance patient survival rates.

In addition, in the clinical use of SII, patients’ histories of comorbidities, as well as their inflammatory habits, such as long-term smoking, should also be taken into consideration.

## Figures and Tables

**Table 1:** Demographic and clinical characteristics of the patients.

	N = 523
Age, *years*	63.5 ± 9.3
Gender, *female*	211 (40.3)
Duration of DM, *years*	
<5 years	18 (3.4)
5–10 years	103 (19.7)
>10 years	402 (76.9)
Insulin use status in DM, *yes*	362 (69.2)
Hypertension, *yes*	489 (93.5)
Duration of hypertension, *years*	
≤5 years	37 (7.6)
>5 years	452 (92.4)
Stroke, *yes*	76 (14.5)
Heart failure, *yes*	145 (27.7)
Lung pathology, *yes*	25 (4.8)
Smoking status, ≥20 *packets/year*	265 (50.7)
Continued smoking in the last 10 Years, *yes*	133 (25.4)
Antihyperlipidemic/antitriglyceridemic drug use, *yes*	364 (70.3)
SII	821.4 ± 1010.8
C-Reactive Protein, *mg/dL*	1.9 ± 6.4

Mean ± Standard deviation, n (%), SII: Systemic immune inflammation index.

**Table 2:** Patients’ first year and following three years’ health outcomes and complication values.

	The First-Year	The Following Three Years	*p*
Polyneuropathy, *yes*	206 (39.4)	179 (43.3)	**<0.001**
Retinopathy progression, *yes*	106 (20.3)	119 (28.8)	**<0.001**
Coronary artery disease, *yes*	154 (29.4)	88 (21.3)	0.555
Acute-chronic renal failure, *yes*	252 (48.2)	207 (50.1)	**<0.001**
Peripheral artery disease, *yes*	138 (26.4)	96 (23.2)	0.362
Hospitalization for any reason, *yes*	363 (69.4)	310 (75.1)	**<0.001**
Hospitalization for DM, *yes*	39 (7.5)	34 (8.2)	0.291
Surgery for Retinopathy, *yes*	522 (99.8)	386 (93.5)	**<0.001**
Death, *yes*	108 (20.7)	29 (7.0)	**<0.001**
Microvascular Complications, *yes*	390 (74.6)	323 (61.8)	**<0.001**
Macrovascular Complications, *yes*	240 (45.9)	159 (30.4)	**<0.001**
Micro + Macrovascular complications, *yes*	201 (38.4)	142 (27.2)	**<0.001**
Microvascular Complications + Death, *yes*	91 (17.4)	27 (5.2)	**<0.001**
Macrovascular Complications + Death, *yes*	73 (14.0)	23 (4.4)	**<0.001**
Micro + Macrovascular complications + Death, *yes*	61 (11.7)	22 (4.2)	**<0.001**

**Table 3:** Comparison of SII values according to micro and macro complications, hospitalization for any reason, and hospitalization for DM parameters.

		Polyneuropathy	Coronary Artery Disease	Retinopathy Progression	Acute-Chronic Renal Failure	Peripheral Artery Disease	Hospitalization for Any Reason	Hospitalization for DM
The first-year	No	803 ± 1151	772.4 ± 692	824.7 ± 1050.9	706.5 ± 566.9	756.4 ± 668.5	670.4 ± 490.3	833.1 ± 1041.8
	Yes	849.6 ± 747	938.6 ± 1521.2	808.2 ± 838.8	944.9 ± 1322.7	1002.6 ± 1610.9	887.9 ± 1163.2	675.4 ± 463.8
	*p*	0.076	0.124	0.997	**0.001**	**0.006**	**0.018**	0.474
The following three years	No	700.4 ± 562	712.4 ± 584.1	739.8 ± 609.7	680.7 ± 553.5	711.9 ± 609.5	609.7 ± 361.8	744.7 ± 610.6
	Yes	770.5 ± 633.2	798.7 ± 629	708.6 ± 555.8	780.6 ± 629.5	793.1 ± 539	771 ± 648.9	576.2 ± 331.7
	*p*	0.119	0.085	0.831	**0.016**	**0.012**	**0.016**	0.139

Mean ± Standard deviation.

**Table 4:** Comparison of SII values according to microvascular and macrovascular complications and death.

		Microvascular Complications	Macrovascular Complications	Death	Micro + Macrovascular Complications	Microvascular Complications + Death	Macrovascular Complications + Death	Micro + Macrovascular complications + Death
The first-year	No	640.5 ± 471.1	736.5 ± 639.8	729.9 ± 593.1	725.7 ± 1122.6	735.6 ± 596.4	747.4 ± 606.5	746.7 ± 602.6
	Yes	883.0 ± 1131.6	921.4 ± 1315.2	1172.9 ± 1861.7	820.1 ± 1422.2	1228.1 ± 2004.8	1277.4 ± 2206.4	1386.8 ± 2393.7
	*p*	**<0.001**	**0.006**	**<0.001**	**0.001**	**<0.001**	**0.003**	**0.001**
The following three years	No	912.2 ± 1423.9	831.4 ± 1145.8	723.2 ± 582.6	821.8 ± 1122.6	818.4 ± 1023.3	815.4 ± 1020.1	814.3 ± 1019.4
	Yes	765.1 ± 628.4	798.2 ± 598.3	844.4 ± 737.7	820.1 ± 620.9	874.2 ± 755.4	951.7 ± 786.9	982.1 ± 791.4
	*p*	0.772	0.195	0.661	0.081	0.718	0.306	0.175

Mean ± Standard deviation.

**Table 5:** ROC analysis results of SII values regarding microvascular and macrovascular complications and death.

		AUC	*p*	Cut-Off	Sensitivity (%)	Specificity (%)
The first-year	Microvascular Complications	0.608	**<0.001**	>540.5	64.1	57.9
	Macrovascular Complications	0.570	**0.005**	>467.6	75.4	37.8
	Death	0.618	**<0.001**	>994.0	35.2	84.8
	Micro + Macrovascular complications	0.589	**<0.001**	>531.1	68.7	47.2
	Microvascular Complications + Death	0.622	**<0.001**	>634.2	64.8	56.3
	Macrovascular Complications + Death	0.607	**0.004**	>1019.2	35.6	84.2
	Micro + Macrovascular complications + Death	0.629	**0.001**	>594.0	73.8	49.4
The following three years	Microvascular Complications	0.508	0.776	>1019	84.5	23.5
	Macrovascular Complications	0.536	0.192	>467.6	74.2	34.3
	Death	0.524	0.711	>838.5	37.9	77.8
	Micro + Macrovascular complications	0.550	0.080	>657.7	52.8	58.3
	Microvascular Complications + Death	0.521	0.757	>838.5	40.7	74.2
	Macrovascular Complications + Death	0.563	0.371	>852.2	43.5	75.0
	Micro + Macrovascular complications + Death	0.585	0.223	>852.2	45.5	75.1

**Table 6:** Multiple linear regression analysis results for predictors of Systemic Immune Inflammation (SII).

Variable	B	*p*-Value
Hypertension	−132.996	0.292
At least 20 pack-years of smoking	180.053	**0.034**
Antihyperlipidemic and antitriglyceridemic drug use	2.806	0.967
DM insulin usage	11.876	0.861
Gender	−22.822	0.758

**Table 7:** Spearman correlation analysis results of SII and CRP in type 2 diabetic retinopathy patients.

		SII	CRP
SII	r	1.000	0.266
	*p*		<0.001
CRP	r	0.266	1.000
	*p*	<0.001	

r: Spearman’s correlation coefficient. Values range between +1 and −1: +1 indicates a perfect positive correlation; −1 indicates a perfect negative correlation; and 0 indicates no correlation. *p*: Statistical significance value: *p* < 0.05 indicates that the correlation is significant at the 95% confidence level; *p* < 0.01 indicates significance at the 99% confidence level. CRP: C-Reactive protein, mg/dL; N: Sample size, which is 523 in this study.

## Data Availability

Our study data contain personal information of patients and, therefore, are not available for sharing due to the ‘Personal Data Protection Law’ and ethical reasons.
